# Simultaneous Talocalcaneal and Talonavicular Joint Dislocation With Navicular Bone Fracture of a Teenager While Playing Football: A Report of a Rare Case

**DOI:** 10.7759/cureus.47848

**Published:** 2023-10-28

**Authors:** Mahmut Gorkem Gurcinar, Muhammed Yusuf Afacan, Sinan Ustundag, Fecri Ciftlik

**Affiliations:** 1 Department of Orthopaedics and Traumatology, Istanbul University-Cerrahpasa, Cerrahpasa Medical Faculty, Istanbul, TUR; 2 Department of Orthopaedics and Traumatology, Istanbul Gelisim University, Istanbul, TUR; 3 Department of Orthopaedics and Traumatology, Medilife Hospital, Istanbul, TUR

**Keywords:** subtalar joint dislocation, talocalcaneal joint dislocation, talonavicular joint dislocation, navicular bone fracture, short leg splint, conservative treatment, teenager sports injury

## Abstract

Simultaneous talocalcaneal and talonavicular joint dislocation, in other words, subtalar joint dislocation, and navicular bone lateral process fracture are rare orthopedic injuries. In this case, we aimed to discuss the effectiveness and ergonomics of non-surgical follow-up with a short leg splint after reduction of talonavicular, talocalcaneal joint dislocation, and lateral process fracture of the navicular bone. A 17-year-old male patient was admitted to the emergency department with swelling and pain in his left foot after spraining his left foot while playing football. Pain, swelling, and deformity in the left foot were evident without a neurovascular deficit. Radiographs showed simultaneous left foot talonavicular joint dislocation, talocalcaneal joint dislocation, and navicular lateral process fracture. The patient underwent closed reduction and a short leg splint. We followed the patient regularly, removed the leg splint in the fourth week, and started various movement exercises. We started muscle strengthening exercises in the sixth week and reached full range of motion with full muscle strength without any deformity in the eighth week. In this case, closed reduction and short leg splint with traction along the axis of the left foot and manipulation of the talus laterally by everting the ankle were sufficient. We restricted the movement of the ankle and tarsometatarsal joint with a short leg splint and reached full range of motion with follow-ups and exercises.

## Introduction

Subtalar joint dislocation is the simultaneous dislocation of the talocalcaneal and talonavicular joints without fractures of the talus and tibia. Subtalar dislocations are generally seen because of high-energy injuries and are rare, isolated injuries, constituting 1% of all traumatic injuries of the foot [1]. We mostly encounter it after high-energy injuries. The most common type is medial dislocation, seen as inversion/rotation injuries. Approximately 80% of them occur as medial dislocations. Lateral dislocations are generally the result of high-energy trauma and occur in around 17% [2]. Injury mechanisms are motor vehicle accidents (38%), falling from heights (30%), and sports injuries (21%). Less frequently, low-energy injuries like falling into a ditch or missing a step may be the cause. While we encounter open injury in 25%, lateral dislocations are more likely to be open injuries. Talonavicular dislocation, talar head fractures, posterior talus fractures, and cuboid and anterior calcaneus fractures may accompany the injury [2]. Pain, stiffness, and loss of motion in the subtalar and ankle joints were observed in the early period. Posttraumatic arthrosis is mostly encountered in the late period. Avascular necrosis and deep infection can also be seen in lateral dislocations [3]. In this study, we wanted to show that a normal range of motion is established and improved after rehabilitation without complications by non-surgical treatment of subtalar joint dislocation. We also emphasized the urgency of preserving anatomical alignment by closed reduction as soon as possible after the trauma and stabilizing the joint with a short leg splint because such injuries may easily be overlooked and the instability may lead to surgery.

## Case presentation

**Figure 1:**
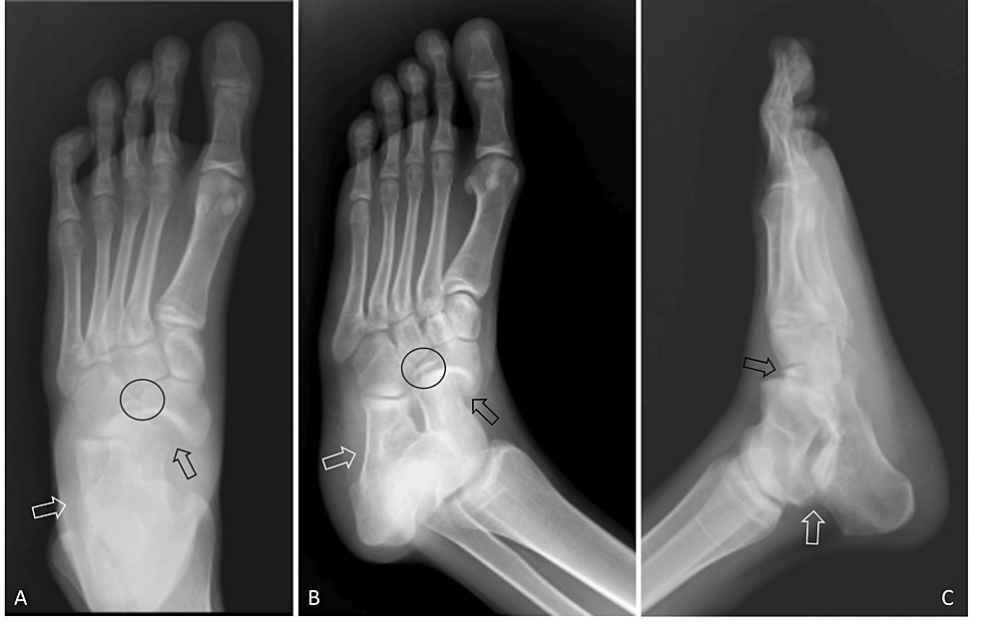
Foot radiographs of the patient before the reduction A: Foot anteroposterior view of the patient before the reduction, showing simultaneous talonavicular, talocalcaneal joint dislocation, and navicular bone lateral process fracture. B: Foot oblique view of the patient before the reduction, showing simultaneous talonavicular, talocalcaneal joint dislocation, and navicular bone lateral process fracture. C: Foot lateral view of the patient before the reduction, showing simultaneous talonavicular, talocalcaneal joint dislocation, and navicular bone lateral process fracture. The white arrow indicates talocalcaneal dislocation, the black arrow indicates talonavicular dislocation, and the circle points out the navicular bone lateral process fracture.

A 17-year-old male was admitted to the emergency department with left foot pain. Swelling, tenderness, and limitation of movement in the lateral aspect of his left foot were evident. He suffered a sprain after slipping on the ball while playing football and was brought to the emergency department immediately. On physical examination, the lateral dorsum of the foot appeared slightly swollen, and the foot was in an inversion position. There was tenderness in the dorsum of the foot upon palpation, and foot joint movements were limited. There was no neurological or vascular deficit. Anteroposterior, lateral, and oblique radiographs revealed left foot medial subtalar joint dislocation (talonavicular and talocalcaneal joint dislocation), and navicular lateral process fracture (Figure 1). 

**Figure 2:**
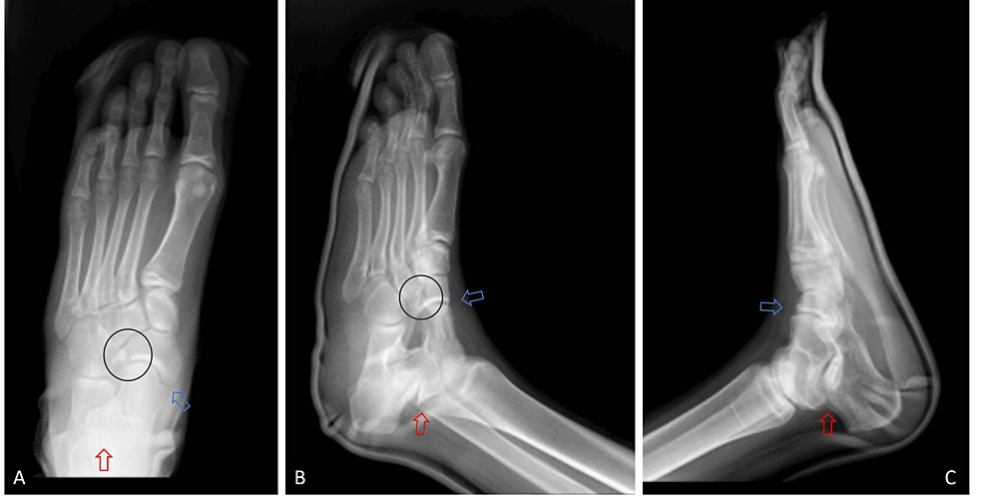
Foot radiographs of the patient after the reduction with short leg splint A: Foot anteroposterior view of the patient after the reduction with short leg splint, showing reduced talonavicular, talocalcaneal joints, and navicular bone lateral process fracture. B: Foot oblique view of the patient after the reduction with short leg splint, showing reduced talonavicular, talocalcaneal joints, and navicular bone lateral process fracture. C: Foot lateral view of the patient after the reduction with short leg splint, showing reduced talonavicular, talocalcaneal joints, and navicular bone lateral process fracture. The red arrow indicates a reduced talocalcaneal joint, the blue arrow indicates a reduced talonavicular joint, and the circle points out the navicular bone lateral process fracture.

In the emergency department, we flexed the patient's knee to relax the crural muscles in the posterior compartment. We performed closed reduction with traction on the foot and manipulation of the talus laterally by forcing the ankle to eversion. A posterior short-leg splint was applied with the ankle at 90 degrees to stabilize and immobilize the ankle, talocalcaneal, talonavicular, and tarsometatarsal joints. Radiographic imaging after the maneuver showed a successful reduction with proper anatomical alignment without neurological and vascular deficit (Figure 2). 

We explained ice application for 20 minutes every two hours, if possible, elevation, appropriate analgesic treatment with paracetamol per oral 3x500 mg if the patient felt pain, follow-up of neurovascular status, and orthopedics and traumatology outpatient clinic follow-up. We followed the patient weekly to check the adequacy of the closed reduction and short leg splint. In the 4th week, we removed the leg splint and started various movement exercises, partial loads, and rehabilitation. In the sixth week, foot and ankle movements regained their normal range of motion. In the sixth week, we started strengthening exercises and allowed full weight bearing. In the eighth week of our follow-up, the patient exhibited a full range of motion without any deformity and could mobilize with full weight bearing and muscle strength.

## Discussion

Subtalar joint dislocation is the occurrence of the simultaneous dislocation of the talocalcaneal and talonavicular joints, while the tibiotalar and calcaneocuboid joints are preserved. Subtalar dislocation occurs because of high-energy injuries and is very rare, making up 1% of all traumatic injuries to the foot [1,2]. The strong ligaments connecting the talus and calcaneus, the biomechanical properties of the ankle, and the tight joint capsule are involved in the rarity of this injury [3].

Subtalar dislocation often occurs in the third decade of life and is ten times more common in men than women [4]. Most cases of subtalar dislocation occur during a fall from a height, a traffic accident, or a basketball or football match [5]. In our patient, it happened due to the foot being forced into inversion after the ball slipped while playing football. Eversion and inversion forces applied to the foot during an accident or fall cause the talonavicular and talocalcaneal ligaments to rupture. The calcaneonavicular ligaments are strong and resist disruption. Eversion and inversion forces make the calcaneus, navicular, and distal bones shift medially or laterally as a unit [6].

Subtalar dislocation should be distinguished from other injuries with a proper clinical examination and radiography. The physical examination shall document any open wound and neurovascular deficit. The patient typically presented with foot and ankle pain and an inability to step forward. Depending on the injury mechanism, the foot's position can be seen in inversion or eversion and an obvious deformity can be seen. However, soft tissue edema may sometimes be encountered in subtalar dislocations without deformity. Thus, such injuries may be overlooked [7]. Our patient's foot was in an inversion position, and he could not step forward. He described severe foot and ankle pain. The inversion position of the foot with severe pain led us to the correct diagnosis. 

If radiographs are normal and the joint is unstable after closed reduction, computed tomography may be required. Regardless of the reduction method, even if the joint is stable after closed reduction, the joint should be fixed with a short leg splint or cast for ligament healing, and no weight bearing is recommended, as this will delay healing [8]. Some authors also recommend percutaneous K-wire fixation for joint stability [2]. We preferred to fix the joint with a short leg splint and did not recommend any weight bearing after closed reduction. We achieved excellent clinical and radiological results without the need for surgery. The patient was able to return to sports in the eighth week.

## Conclusions

This case report briefly illustrated a simultaneous talonavicular and talocalcaneal joint dislocation and navicular bone lateral process fracture, which are rare orthopedic combination injuries requiring rapid and correct diagnosis. Since this trauma can be easily overlooked, the fact that the patient's foot is in an inversion position helps guide the diagnosis in this case. The closed reduction was successful due to being performed promptly. The easily applicable maneuver was the longitudinal traction of the foot with the knee in flexion and with manipulation of the talus laterally by everting the foot. A short leg splint from the posterior preserved stabilization after reduction and immobilized navicular bone for fracture healing without the requirement for surgical intervention. Due to the timely rehabilitation, we achieved a full range of motion and full weight bearing with full muscle strength.
